# Therapeutic effect of *Tripterygium wilfordii* Hook F multiglycosides on gut barrier dysfunction in rats with acute necrotizing pancreatitis

**DOI:** 10.3892/etm.2012.817

**Published:** 2012-11-19

**Authors:** JIE WANG, GANG WU, BAOJIN MA, JIANHUA WU, DUAN CAI

**Affiliations:** Department of Surgery, Huashan Hospital, Fudan University, Shanghai, P.R. China

**Keywords:** acute necrotizing pancreatitis, gut barrier dysfunction, *tripterygium wilfordii* hook f multiglycosides, therapy

## Abstract

The aim of the current study was to investigate the therapeutic effect of *Tripterygium wilfordii* Hook F multiglycosides (TWG) on gut barrier dysfunction in rats with acute necrotizing pancreatitis (ANP). ANP was induced in rats using 3.5% sodium taurocholate. The rats were divided into 3 groups: the sham operation (SO), ANP and ANP+TWG groups. Biochemical and pathological change of pancreatic tissue and ileal mucosa, bacterial cultures and the survival rate were measured following surgery and treatment. TWG treatment significantly decreased amylase and lipase activities and plasma endotoxin and D-lactate levels. Edema and inflammation in the pancreas and ileal mucosa were alleviated. Positive bacterial cultures were significantly reduced. The survival rate of the rats in the ANP+TWG group was higher than that of the rats in the ANP group. TWG treatment showed beneficial effects by protecting the pancreas from bacterial infection caused by gut barrier dysfunction and improving the outcomes of the rats with ANP.

## Introduction

Early multiple organ failure (MOF) and subsequent infection are serious complications of acute necrotizing pancreatitis (ANP) and are the leading cause of mortality in ANP patients, accounting for >80% of the total ANP mortality (1). Damage to the intestinal barrier function (IBF) and bacterial translocation (BT) are currently considered the most likely sources of pathogens. The gut also plays a role in priming neutrophils and the release of inflammatory cytokines, which initiate and propagate nearly all the detrimental consequences of severe inflammation and sepsis. Future research approaches and potential therapeutic measures that may restore and preserve gut barrier function are being explored (2).

*Tripterygium* has clear anti-inflammatory and immunomodulatory effects, and inhibits the excessive release of inflammatory mediators at multiple levels (3). In the current study, *Tripterygium wilfordii* Hook F multiglycosides (TWG) were used to treat a rat model of ANP. During the treatment, the mechanism of TWG on the treatment of intestinal mucosal barrier dysfunction caused by ANP was investigated through observation of serum amylase levels, lipase activity, plasma endotoxin levels, organ bacterial cultures, plasma D-lactate concentrations, pathological changes of the pancreas and intestinal mucosa and the survival rates of the rats.

## Materials and methods

### 

#### Animals and pancreatitis model

SD rats (male, 10–12 weeks old, weighing 200–250 g) were fasted but allowed to drink water freely for 16 h before the experiment. They were allocated randomly to one of three groups: the sham operation (SO), ANP and ANP+TWG groups. The SO group rats (n=12), underwent laparotomy under general anesthesia, as described for the ANP group, and sham intubation of the cholo-pancreatic duct but without any drug injection. These rats were sacrificed 24 h later. In the ANP group, laparotomy was performed through a midline incision. After cannulation of the common biliopancreatic duct with a 28-gauge, 0.5-inch microfine catheter, a microaneurysm clip was placed on the bile duct below the liver and another around the common biliopancreatic duct at its entry into the duodenum to avoid reflux of enteric contents into the duct. Then, 2 ml/kg 3.5% sodium taurocholate (Sigma, St. Louis, MO, USA) was slowly infused into the common biliopancreatic duct at a volume of 2.0 ml/kg at 0.25 ml/min. The infusion pressure was kept <3.4–3.9 kPa, as measured using a mercury manometer. When the infusion was complete, the two microclips were removed and the abdomen was closed in two layers. All procedures were performed using sterile techniques. The ANP+TWG group underwent the induction of acute pancreatitis and treatment with TWG. After pancreatitis was induced, as described for the ANP group, the rats were injected intraperitoneally 60 min later with TWG 50 mg/kg. Six animals in each group were randomly selected for observation of survival. The other animals were sacrificed 24 h after surgery.

#### Amylase and lipase measurement

Blood samples were drawn from the aorta 8 h after the induction of ANP, and serum amylase/lipase levels were measured using a standard clinical automated analyzer.

#### Plasma endotoxin

Following laparotomy under sterile conditions, blood samples from the abdominal aorta were acquired and centrifuged to isolate plasma. The endotoxin activities of the plasma were determined using a Limulus amebocyte lysate (LAL) assay kit (QCL-1000, catalog no. 50-647U; BioWhittaker, Shanghai, China) according to the manufacturer’s instructions. Two different charges of elastase preparations were tested.

#### Quantitative cultures and bacterial identification

Following laparotomy under sterile conditions, blood samples from the abdominal aorta and ascites were acquired and put into sterile tubes. Mesenteric lymph nodes (MLNs), one portion of the pancreas, livers and lungs were harvested, put into anaerobic chambers, and processed for the culturing of aerobic and anaerobic organisms using a standardized method. Each sample was weighed and homogenized. Afterwards, the homogenates were diluted serially, quantitatively plated in duplicate on phenylethyl alcohol and MacConkey II agar, and then incubated aerobically at 37°C for 24 h. Bacterial counts were expressed as colony-forming U/g tissue, and counts of ≥1,000 colony-forming U/g were considered to represent a positive culture. Gram-negative bacteria were identified using the API-20E system (bioMerieux Vitek, Hazelwood, MO, USA). Gram-positive bacteria were identified to the genus level using standard microbiological methods.

#### D(-)-lactate determination

The plasma from systemic blood samples was obtained and subjected to a deproteination and neutralization process by acid/base precipitation using perchloric acid and potassium hydroxide. The protein-free plasma was then assayed for D(-)-lactate concentration by a previously described enzymatic-spectrophotometric method with minor modification (4).

#### Histopathologic analysis

A portion of the pancreas from the same anatomical location in each rat, including the main pancreatic duct, was fixed in 10% neutral-buffered formalin and embedded in paraffin. One paraffin section stained with hematoxylin and eosin was examined for each pancreas. Two pathologists, who were blinded to the treatment protocol, scored the tissues with respect to edema, acinar necrosis, inflammatory infiltrate, hemorrhage, fat necrosis and perivascular inflammation in 20 fields. The scores for each histological examination were summed, yielding a maximum score of 24, as defined by Schmidt *et al*(5).

#### Electron microscopy

The relationship between zymogen granules and autophagic vacuoles was examined in the acinar cells of rats 5 h after duct ligation using transmission electron microscopy. From the freshly excised pancreas, small cubes <1 mm^3^ in size were sliced and immediately fixed in Karnovsky’s fixative (2.5% glutaraldehyde, 4% paraformaldehyde and 1 M Na cacodylate buffer, pH 7.4). The samples were post-fixed in 1% osmium tetroxide, dehydrated in graded concentrations of ethanol, incubated in ascending concentrations of propylene oxide and embedded in Spurr’s epoxy resin. Ultrathin sections were stained with uranyl acetate and bismuth subnitrate and viewed under a Hitachi transmission electron microscope (H-7000) at Fudan University.

#### Statistical analysis

The results are expressed as the mean ± standard error of the mean. Translocation incidence was evaluated by Fisher’s exact test. The significance of differences in total histopathologic scores, serum amylase activities and cytokine levels were assessed using one-way analysis of variance and Tukey’s HSD as post hoc tests. Detailed histo-pathological scores (e.g., for edema and acinar necrosis) were assessed using the Kruskal-Wallis test, and subgroup analyses were conducted using the Mann-Whitney U test. P<0.05 was considered to indicate a statistically significant result. All statistical measurements were performed using SPSS PC version 9.05 (SPSS, Inc., Chicago, IL, USA).

## Results

### 

#### Survival rates of the ANP and ANP+TWG groups

The majority of the animals in the ANP group died in the first 3 days after the induction of pancreatitis: at the 3-day interval, 5 of 6 rats had died, corresponding to a death rate of 83.3%. By contrast, TWG led to a significant reduction of the death rate, with only 2 deaths in 6 rats (33.3%). Autopsy of the rats that died during the experiment revealed extensive intra- and extrapancreatic necrosis, pancreatic hemorrhage and moderate to massive hemorrhagic ascites in animals who died within 48 h after the induction of pancreatitis. At later time intervals, the upper abdomen had changed to an inflammatory mass harboring multiple abscess formations, which caused severe intestinal obstruction in certain cases. In the lungs, exudative congestion was observed, whereas the liver and kidneys appeared grossly normal.

#### Plasma amylase and lipase levels

Compared with baseline values, all rats with pancreatitis showed increased lipase and amylase levels (P<0.01). However, the amylase and lipase concentrations were found to be decreased (P<0.01) in the ANP+TWG group (Table I).

#### Plasma endotoxin

The plasma endotoxin level of the ANP group was 0.067±0.012 EU/ml, which was significantly higher than that of the SO group (0.0330±0.007 EU/ml; P<0.01). However, the endotoxin levels in the TWG treated group (0.052±0.014 EU/ml) were significantly lower than those in the ANP group (P<0.01; Table I).

#### Bacterial cultures

The results of bacterial culture were negative for the SO group. The positive rate of the ANP group was 43.1%, in which the highest positive rate (75%) was obtained for the ascites bacterial culture (Table II). However, with the exception of MLNs, the positive rate of the ANP+TWG group was significantly lower than that of the ANP group (P<0.05). The most common bacteria identified in the ANP and ANP+TWG groups were *Enterococcus* sp. and *Proteus* sp. (Table III).

#### Changes in plasma D(-)-lactate

The level of D(-)-lactate in the systemic blood of the SO group was lower than that of the ANP group. Following TWG treatment, the level of D(-)-lactate declined significantly (Table IV).

### Changes in histopathology

#### Pancreas

**Figure 1.**
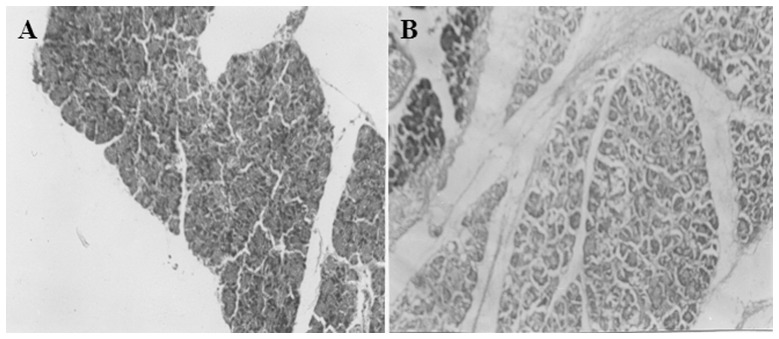
Light microscopy observation of rat pancreas (magnification, ×40). (A) The ANP group exhibited clear pancreatic interstitial hyperemia and edema, widened lobular intervals and vascular congestion; (B) The ANP+TWG group exhibited acinar necrosis, hemorrhage and fat necrosis similar to that in the ANP group, but the signs of pancreatic edema and inflammatory cell infiltration in ANP+TWG group were reduced compared with those in the ANP group. ANP, acute necrotizing pancreatitis; TWG, *Tripterygium wilfordii* Hook F multiglycosides.

The following features were observed in the pancreas specimens: i) General observation: for the ANP group, extensive bloody intra-abdominal ascites, widening of the ducts and saponification spots around the pancreas and mesentery were observed, the volume of the pancreas increased, and congestion and edema occurred with hemorrhage and necrosis. The degree of pathological change in the ANP+TWG group was milder than that in the ANP group at the same time interval. ii) Light microscopy observation: in the ANP group (Fig. 1A), pancreatic interstitial hyperemia and edema, widening of lobular intervals, including acinar intervals, vascular congestion, interstitial hemorrhage and saponified or extensive acinar necrosis occurred, accompanied by extensive monocyte and neutrophil infiltration. Fat necrosis with saponification spots also occurred. In the ANP+TWG group, acinar necrosis, hemorrhage and fat necrosis were similar to those in the ANP group. However, the degree of pancreatic edema and inflammatory cell infiltration in the ANP+TWG group was reduced (Fig. 1B), compared with that in the ANP group at the same time interval. iii) Pathological evaluation: the levels of congestion, edema and necrosis in the ANP group were significantly higher than those in the SO group. In the ANP+TWG group, the pathological features were clearly reduced (Table V).

#### Ileal mucosa

**Figure 2.**
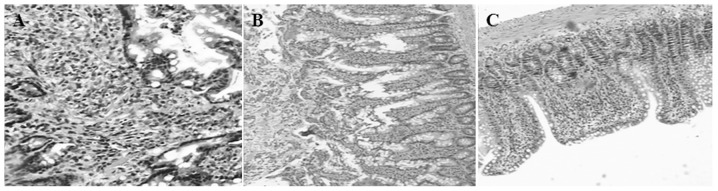
Light microscopy observation of rat ileal mucosa (magnification, ×40). (A) The mesenchymal layer of the ileal epithelium in the ANP group revealed marked edema with inflammation. (B) The villi of the ileal epithelium in the ANP group were disorderly, and necrosis and shedding were observed. (C) The ileal epithelium of the ANP+TWG group was orderly, and hyperplasia of the villous epithelium occurred. ANP, acute necrotizing pancreatitis; TWG, *Tripterygium wilfordii* Hook F multiglycosides.

**Figure 3.**
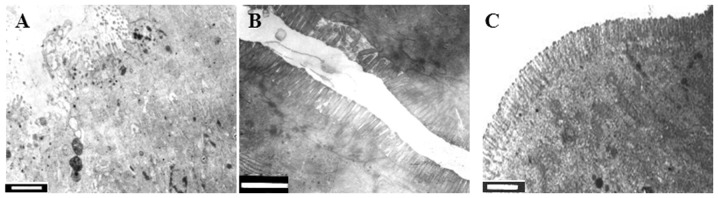
Electron microscopy observation of rat ileum. (A) In the SO group, cells and organelle retained their integrity, and microvilli were arranged in a compact and orderly manner (scale bar, 1 μm). (B) In the ANP group, cellular edema occurred, granules in the cells were loose and some mucosal fracture was observed (scale bar, 2 μm). (C) In the ANP+TWG group, the granules in the cells were slightly loose but the organelles retained their integrity (scale bar, 1 μm). SO, sham operation; ANP, acute necrotizing pancreatitis; TWG, *Tripterygium wilfordii* Hook F multiglycosides.

The following featues were observed in the ileal mucosa: i) Light microscopy observation: for the ANP group, there was marked congestion, edema, lymphatic expansion and villous thickening, together with monocyte infiltration and inflammation, columnar epithelial cell necrosis and loss, some loss of villi and damage in the mesenchymal layer of the ileal mucosa (Fig. 2A and B). The lesions in the ANP+TWG group were clearly reduced in severity compared with those in the ANP group. Active epithelium hyperplasia of the intestinal mucosa was observed in the ANP+TWG group (Fig. 2C). ii) Electron microscopic analyses: cells were compact, microvilli were arranged compactly and neatly, and organelles maintained their integrity in the SO group (Fig. 3A). By contrast, in the ANP group, marked edema, loose particles and organelle damage in the cells, disordered and necrotic microvilli and damaged mucosa were visible (Fig. 3B). However, slightly loose particles and almost compact organelles were observed in the ANP+TWG group (Fig. 3C). iii) Villous height and mucosal thickness were clearly reduced in the ANP group, but in the TWG treatment group, the pathological changes observed in the ANP group were alleviated significantly (Table VI).

#### Correlation between plasma D(-)-lactate concentration and villous height and mucosal thickness

**Figure 4.**
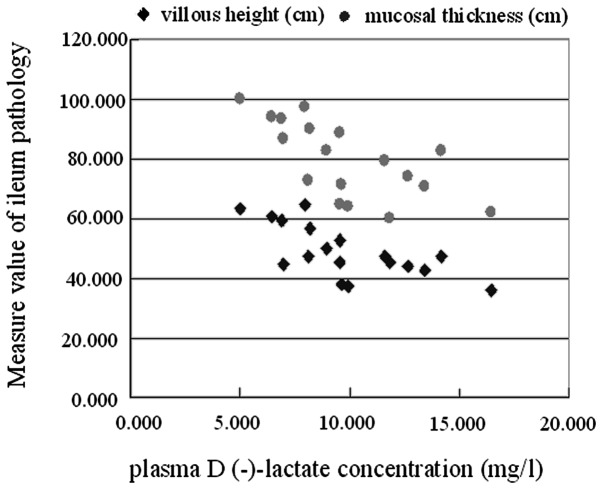
Correlations between plasma D(-)-lactate concentration and villous height and mucosal thickness. Plasma D(-)-lactate concentration showed significant negative correlations with villous height and mucosal thickness.

The correlations between plasma D(-)-lactate concentration and villous height and mucosal thickness were examined using regression analysis. Plasma D(-)-lactate concentration and villous height showed a significant negative correlation (r=−0.684, P<0.01), as did plasma D(-)-lactate concentration and mucosal thickness (r=−0.677, P<0.01; Fig. 4).

## Discussion

Changes in the permeability of the intestinal mucosa may be manifested through alterations in gastrointestinal pathology, intestinal microbes and metabolites, obstacles in IgA synthesis and secretion and endotoxemia. Plasmid-labeled *E. coli* have demonstrated that intestinal bacteria are able to pass through the intestinal mucosa to reach the mesenteric lymph nodes, and to reach other remote organs via the blood in ANP (6).

Nettelbladt *et al* and Cicalese *et al*(7,8) reported that labeled bacterial strains may be detected at remote organs after feeding labeled bacteria to ANP mice, and the occurrence of bacterial translocation (BT) was 100%. During ANP, the blood flow in the body is redistributed; blood flow in the gastrointestinal tract is reduced, which results in intestinal mucosal ischemia and hypoxia. Intestinal microcirculation blood flow declines within 15 min of ANP. A study by Lemaire *et al*(9) found that following ischemia of the intestinal mucosa for 30–40 min, epithelium cells stop the synthesis of protective glycoprotein and absorb endotoxin before pathological damage occurs. During ANP, large amounts of cytokines and inflammatory mediators are released. Among them, TNF-α, IL-β, IL-8 and leukotriene may increase ischemia and injury of the intestinal mucosa. The ‘two-hit’ theory suggests that leukocytes which perfuse to various tissues are activated by pro-inflammatory factors that are released by macrophages during ANP; the activated leukocytes release a large number of protein decomposition enzymes, oxygen free radicals and cytokines, causing an excessive systemic immune inflammatory response and further aggravating the injury of the intestinal mucosa, eventually forming a vicious cycle of damage-increased permeability-further damage (10). There is a significantly positive correlation between gut hyper-permeability and the severity of pancreatitis. An increase of intestinal permeability indicates gut barrier dysfunction (11). Though these potential mechanisms, ANP eventually leads to disturbances of the microcirculation, ischemia and increases of vascular permeability, impairment of the intestinal mucosal barrier, and translocation of intestinal bacteria and endotoxins into the blood. At the same time, products of bacterial metabolism, including D-lactic acid, are absorbed into the blood, further increasing the systemic inflammatory response.

D-lactic acid is the one of metabolism products of inherent intestinal bacteria, including *E. coli, Lactobacillus* and *Klebsiella*. Mammalian liver is lacking in the enzymes that are able to break down D-lactic acid. D-lactic acid passes into blood via the portal vein, and leads to a concentration change. Therefore, plasma D-lactate concentrations in the inferior vena cava may reflect the permeability of the intestinal tract. Murry *et al*(12) found that the D-lactic acid concentrations in patients with intestinal ischemia were significantly increased among patients undergoing laparotomy. Another report showed that the D-lactate concentration correlated significantly with the intestinal mucosal injury score in critical patients with intestinal ischemia, intestinal ischemia-reperfusion and burns (13). It was also synchronized with the level of endotoxin, and increased significantly within 1–1.5 h after ANP. Therefore, D-lactic acid is an early warning signal for intestinal mucosa dysfunction, and may be detected simply to monitor intestinal dysfunction in critical illness. Ammori *et al* used the kidney excretion ratio of oral polyethylene glycol 3350 to polyethylene glycol 400 to investigate the permeability of the intestine in ANP (14). The authors found that the ratio in the patients who developed MOF and/or succumbed was much higher than that in other critically ill patients, which suggests that the early increase in intestinal permeability of patients with severe acute pancreatitis is likely to have a significant impact on pathophysiological changes.

In the current study, light microscopy in the ANP group showed interstitial edema and a small amount of bleeding in the ileum, expansion of lymph ducts and vessels, neutrophil infiltration with lymphoid follicular hyperplasia, villous edema, thickening, height reduction, arrangment disorder and necrosis and thinning of the mucosa. Electron microscopy showed marked edema in the cells, granular loosening, rupture of organelles, disordered microvilli, large areas of necrosis and detachment, and damage in areas of the mucosa. These morphological changes indicate the mechanical damage of the intestinal barrier. The increase of plasma D-lactate concentration was identified to be significantly correlated with the thickness of the intestinal mucosa, which demonstrates that the plasma concentration of D-lactate reflects the functional damage of the intestinal mucosal barrier in the ANP rats, which provides the conditions for BT. The SO group revealed no such changes, which is consistent with earlier studies (15). Other experiments showed that the endotoxin levels and positive rates of organ bacterial culture were significantly increased in ANP rats. Bacterial culture results revealed that the positive rate in the ANP group was 55.6%; the highest positive rate was observed in ascites 75%. Strain identification revealed that the bacteria most commonly involved were *Enterococcus* species and *Proteus mirabilis*, which demonstrates that endotoxemia and intestinal BT are the result of early increases of intestinal permeability and intestinal barrier damage.

Chinese medicine has been widely used in the treatment of ANP. The clinical application of triptolide is common in traditional Chinese medicine (16). Triptolide is an immunosuppressant which has anti-inflammatory activity and inhibits cell-mediated and humoral-mediated immune function. TWG is a compound that is chemically purified from the root of *Tripterygium* by removing the toxic root skin and other toxic components, which have a high value in original Chinese medicine.

It has been identified that *Tripterygium* has a dual role in immune regulation. Low doses increase the cytotoxic activity of natural killer cells, correct distribution disorders of T subset cells and regulate the immune response. In addition, triptolide itself has a direct anti-inflammatory effect. It inhibits the increase in vascular permeability in inflammation, the chemotaxis of inflammatory cells, the production and release of inflammatory mediators and platelet aggregation. *Tripterygium*, due to its potent anti-inflammatory and immune regulatory effects, is able to inhibit the production of cytokines, including TNF-α, IL-6 and IL-8, and the phagocytic function of phagocytes (17,18). The immunosuppressive, cartilage protective and anti-inflammatory effects of TWG extracts are well documented (19). Our previous studies have shown that *Tripterygium wilfordii* is able to reduce serum endotoxin, TNF-α and IL-1 levels in ANP rats (3). Wang *et al*(20) in another study found that a combination of *Salvia miltiorrhiza* and *Tripterygium wilfordii* was able to significantly reduce serum amylase, inhibit excessive inflammatory cytokine production and reduce the extent of tissue damage in the treatment of ANP.

The presence of large amount of ascites associated with ANP results in intestinal paralysis and weakened gastrointestinal motility, which undermines the resistance of the intestine to bacteria invasion. During ANP, systemic immunity is impaired. Intestinal bacteria and bacterial toxins are able to pass through the mucosa into the submucosa, reach mesenteric lymph nodes and eventually enter distant organs through the blood (2). The current study found that serum endotoxin, serum amylase and lipase activities in the ANP+TWG group were significantly lower than in the ANP group. The plasma D-lactate concentration also decreased in the ANP+TWG group. The positive rates of bacterial cultures from the parenteral organs, blood and ascites in the ANP+TWG group were lower than in the ANP group. Furthermore, the pathology score determined by optical microscopy was improved in the ANP+TWG group. It was demonstrated that TWG reduces the intestinal permeability to endotoxins in ANP, maintains the structure integrity of mucosal cells and has a therapeutic effect on intestinal barrier dysfunction and BT in rats with ANP.

In conclusion, intestinal barrier dysfunction and intestinal BT exist in ANP rats. TWG improves the pancreatic and intestinal damage, strengthens the biological barriers of the intestinal tract, reduces gut-derived bacteria and the incidence of endotoxin translocation, and prevents the development of ANP.

## Figures and Tables

**Table I.** Amylase, lipase and endotoxin levels in different groups of rats.

Group	Sample no.	Amylase (U/l)	Lipase (U/dl)	Endotoxin (EU/ml)
SO	6	5000±580	200±9	0.033±0.007
ANP	12	30000±900	800±220	0.067±0.012
ANP+TWG	12	10000±550^a^	600±90^a^	0.052±0.014^a^

a P<0.01 compared with the ANP group. SO, sham operation; ANP, acute necrotizing pancreatitis; TWG, *Tripterygium wilfordii* Hook F multiglycosides.

**Table II.** Results of bacterial cultures (%).

Group	Sample no.	Ascites	Blood	MLNs	Liver	Pancreas	Spleen
SO	6	0	0	0	0	0	0
ANP	12	9 (75)	2 (17)	6 (50)	8	8 (67)	7 (59)
ANP+TWG	12	4 (34)	1 (9)	2 (17)	0	0	0

SO, sham operation; ANP, acute necrotizing pancreatitis; TWG, *Tripterygium wilfordii* Hook F multiglycosides; MLNs, mesenteric lymph nodes.

**Table III.** Bacterial classifications of the ANP and ANP+TWG groups.

Group	*Enterococcus* sp.	*Proteus* sp.	*E. coli*	*Bacillus*	*Pneumococcus*
ANP	9	7	5	1	-
ANP+TWG	3	1	-	-	-

ANP, acute necrotizing pancreatitis; TWG, *Tripterygium wilfordii* Hook F multiglycosides.

**Table IV.** Comparison of plasma D(-)-lactate concentrations in rats of different groups.

Group	Sample no.	D(-)-lactate concentration (mg/l)
SO	6	9.888±1.008
ANP	12	11.098±2.745^a^
ANP+TWG	12	7.772±2.916^b^

a P<0.01 compared with the SO group;

b P<0.05 compared with the ANP group. SO, sham operation; ANP, acute necrotizing pancreatitis; TWG, *Tripterygium wilfordii* Hook F multiglycosides.

**Table V.** Pathology evaluation of rats with ANP.

Group	Edema	Inflammation	Hemorrhage	Necrosis
SO	0.17±0.02	0.00±0.00	0.00±0.00	0.00±0.00
ANP	3.00±0.00	3.28±0.39	2.89±0.44	2.67±0.33
ANP+TWG	2.39±0.08^a^	2.06±0.74^a^	1.00±0.44^a^	0.84±0.17^a^

a P<0.01 compared with the ANP group. SO, sham operation; ANP, acute necrotizing pancreatitis; TWG, *Tripterygium wilfordii* Hook F multiglycosides.

**Table VI.** Comparison of villous height and mucosal thickness in rats of different groups.

Group	Sample no.	Villous height (μm)	Mucosal thickness (μm)
SO	6	57.25±3.580	90.653±8.026
ANP	12	44.278±6.081^a^	70.833±10.217^a^
ANP+TWG	12	55.597±6.902^b^	87.736±11.794^b^

a P<0.01 compared with the SO group,

b P<0.01 compared with the ANP group. SO, sham operation; ANP, acute necrotizing pancreatitis; TWG, *Tripterygium wilfordii* Hook F multiglycosides.
